# Supplementary material for: The COVID-19 pandemic effect on the prehospital Madrid stroke code metrics and diagnostic accuracy
Source: PLoS One. 2022 Oct 10;17(10):e0275831. doi: 10.1371/journal.pone.0275831 (PMC9550046; doi:10.1371/journal.pone.0275831)
Supplement: S1 Table — (DOCX) [file pone.0275831.s001.docx]

**Supporting Table 1: List of MBDS diagnosis of AS.**

$$\boldsymbol{Diagnostic accuracy=}\frac{\boldsymbol{MBDS diagnosis of AS}}{\boldsymbol{Total number of records}}$$

List of MBDS diagnosis of AS:

| ICD-10 Code | Disease |
| --- | --- |
| G45 | Transient cerebral ischaemic attacks and related syndromes |
| G46 | Vascular syndromes of brain in cerebrovascular diseases |
| I60 | Subarachnoid haemorrhage |
| I61 | Intracerebral haemorrhage |
| I62 | Other nontraumatic intracranial haemorrhage |
| I63 | Cerebral infarction |
| I65 | Occlusion and stenosis of precerebral arteries, not resulting in cerebral infarction |
| I66 | Occlusion and stenosis of cerebral arteries, not resulting in cerebral infarction |
