# Supplementary material for: The COVID-19 pandemic effect on the prehospital Madrid stroke code metrics and diagnostic accuracy
Source: PLoS One. 2022 Oct 10;17(10):e0275831. doi: 10.1371/journal.pone.0275831 (PMC9550046; doi:10.1371/journal.pone.0275831)
Supplement: S1 File — (DOCX) [file pone.0275831.s002.docx]

**Supporting information 1: Members of the Madrid Stroke Network and Ictus Plan of de Community of Madrid (members as of 2021)**

| **Member** | **Affiliation** | **Columna1** |
| --- | --- | --- |
| Blanca Fuentes Gimeno | Department of Neurology | Hospital Universitario La Paz |
| Jorge Campollo Velarde | Department of Radiology | Hospital Universitario 12 de Octubre |
| Roberto García Leal | Department of Neurosurgery | Hospital Universitario Gregorio Marañón |
| Blanca Palomino Aguado | Physical Medicine and Rehabilitation | Hospital Universitario Ramón y Cajal |
| María Isabel Egocheaga Cabello | Primary Care | Centro de Salud Isla de Oza |
| Jorge Gómez Cerezo | Department of Internal Medicine | Hospital Universitario Infanta Sofía |
| Alfonso Martín Martínez | Emergency Department | Hospital Severo Ochoa Leganés |
| Rogelio Simón de las Heras | Department of Pediatric Neurology | Hospital Universitario 12 de Octubre |
| Nicolás Riera López | Emergency Medicine Service | SUMMA 112 |
| Joaquín Carneado Ruiz | Department of Neurology | Hospital Universitario Puerta de Hierro |
| María Alonso de Leciñana Cases | Department of Neurology | Hospital Universitario La Paz |
| Patricia Calleja Castaño | Department of Neurology | Hospital Universitario 12 de Octubre |
| José Egido Herrero | Department of Neurology | Hospital Clínico Universitario San Carlos |
| Antonio Gil Núñez | Department of Neurology | Hospital Universitario Gregorio Marañón |
| Jaime Masjuán Vallejo | Department of Neurology | Hospital Universitario Ramón y Cajal |
| José Vivancos Mora | Department of Neurology | Hospital Universitario La Princesa |
| Aránzazu Vázquez Doce | Department of Physical Medicine and Rehabilitation | Hospital Universitario La Princesa |
| David Hernández Herrero | Physical Medicine and Rehabilitation | Hospital Universitario La Paz |
| Pablo Busca Ostaloza | Emergency Medicine Service | SUMMA 112 |
| Fátima Gutiérrez Sánchez | Emergency Medicine Service | SUMMA 112 |
| Fernando Fortea Gil | Department of Radiology | Hospital Universitario Gregorio Marañón |
| José Luís Caniego Monreal | Department of Radiology | Hospital Universitario de la Princesa |
| José Carlos Méndez Zendón | Department of Radiology | Hospital Universitario Ramón y Cajal |
| Manuel Moreu Gamazo | Department of Radiology | Hospital Universitario Clínico San Carlos |
| Pedro Navia Alvarez | Department of Radiology | Hospital Universitario de la Paz |
| Aurelio Vega Astudillo | Department of Radiology | Hospital Universitario Puerta de Hierro Majadahonda |
| Olga Mateo Sierra | Department of Neurosurgery | Hospital Universitario Gregorio Marañón |
| José Antonio Fernández Alén | Department of Neurosurgery | Hospital Universitario 12 de Octubre |
| Pablo de Andrés Guijarro | Department of Neurosurgery | Fundación Jiménez Díaz |
| Raquel Gutiérrez | Department of Neurosurgery | Hospital Universitario Puerta de Hierro Majadahonda |
| Luis Ley Urzaiz | Department of Neurosurgery | Hospital Universitario Ramón y Cajal |
| Borja Hernández | Department of Neurosurgery | Hospital Universitario La Paz |
| Rebeca Alfayate | Department of Neurosurgery | Hospital Clínico Universitario San Carlos |
| Lucía Alcázar | Department of Neurosurgery | Hospital Universitario de la Princesa |
| Juan Carlos Gómez-Angulo | Department of Neurosurgery | Hospital de Getafe |
