# Supplementary material for: The COVID-19 pandemic effect on the prehospital Madrid stroke code metrics and diagnostic accuracy
Source: PLoS One. 2022 Oct 10;17(10):e0275831. doi: 10.1371/journal.pone.0275831 (PMC9550046; doi:10.1371/journal.pone.0275831)
Supplement: S2 File — Description of the M-Direct scale. (DOCX) [file pone.0275831.s003.docx]

**Supporting information 2: Madrid Direct scale (M-Direct)**

| Item | Scoring |
| --- | --- |
| Motor system: ARM * | **0**: Overcomes gravity even with help  **+1**: Does not overcome gravity |
| Motor system: LEG * | **0**: Overcomes gravity even with help  **+1**: Does not overcome gravity |
| Conjugate gaze * | **0**: Normal  **+1**: Conjugate deviation of gaze to one side |
| Response to orders  or Recognition of the deficit * | **0**: Normal  **+1**: Does not obey orders.  Or does not recognize their weakness or weak side |
| Systolic blood pressure | **0**: Below 180 mmHg  **-1**: 181-190 mmHg  **-2**: 191-200 mmHg  **-3**: 201-210 mmHg  **-4**: 211-220 mmHg |
| Age | If comorbidity, -1 for each year exceeding 85 * |
|  | If TOTAL> 1, direct transfer to centre for thrombectomy * |

* Read the attached notes

**NOTES:**

- In the motor items, only the limb that cannot overcome gravity is scored (a muscle balance of 0 to 2 or a score on the NIHSS scale of 3 or 4 in this item).

- Gaze deviation is scored as either partial or forced (NIHSS of 1 or 2 in this item).

- The response to orders is scored if the patient does not obey half or more than half of simple orders (NIHSS of 1 or 2 in this item).

- The recognition of the deficit is evaluated by asking the patient “whose arm is this?”, or “Is this arm yours?”, as well as “can you move your arms well?”. If either of the two answers is incorrect or the patient shows other very evident signs of not recognizing his deficit, a score of 1 is given. In this case, visual or sensory extinction is not evaluated.

- The response to orders and recognition of the deficit are mutually exclusive items, since it is necessary to obey orders to answer the recognition questions.

- In the case of patients whose score is <2 due exclusively to their age and whose baseline situation is excellent, the possibility of direct transfer can be evaluated with the neurologist of the hospital on call for TM.

- The positive Madrid-Direct scale (2, 3 or 4) indicates direct transfer to a centre with the capacity to perform mechanical thrombectomy.
