# Supplementary material for: The COVID-19 pandemic effect on the prehospital Madrid stroke code metrics and diagnostic accuracy
Source: PLoS One. 2022 Oct 10;17(10):e0275831. doi: 10.1371/journal.pone.0275831 (PMC9550046; doi:10.1371/journal.pone.0275831)
Supplement: S3 File — Manuscrito en Español. (DOCX) [file pone.0275831.s004.docx]

# Estudio cuasiexperimental sobre el efecto de la pandemia por COVID-19 en el protocolo código ictus de la Comunidad de Madrid

Authors:

Nicolás Riera-López ^1^, Andrea Gaetano-Gil ^2^, José Martínez-Gómez ^3^, Nuria Rodríguez-Rodil ^3^, Borja M Fernández-Félix ^2,4^, Jorge Rodríguez-Pardo ^5^, Carmen Cuadrado-Hernández^6^, Emmanuel Pelayo Martínez-González^6^, Alicia Villar-Arias ^6^, Fátima Gutiérrez-Sánchez ^6^, Pablo Busca-Ostolaza ^6^, Eduardo Montero-Ruiz ^7^, Exuperio Díez-Tejedor ^5^, Javier Zamora ^2,4,8&^, Blanca Fuentes-Gimeno ^5&,^ en nombre de la Red Ictus de la Comunidad de Madrid ^^^.

^1^Comisión Ictus. Servicio de Urgencia Médica de Madrid (SUMMA 112). España

^2^Unidad de Bioestadística Clínica. Hospital Universitario Ramón y Cajal. IRYCIS. España

^3^Departamento de informática. Servicio de Urgencia Médica de Madrid (SUMMA 112). España

^4^CIBER de Epidemiología y Salud Pública (CIBERESP). España

^5^Departamento de Neurología y Centro de Ictus. Instituto de investigación IdiPAZ (Hospital Universiterio La Paz, Universidad Autónoma de Madrid). España

^6^ Servicio de Urgencia Médica de Madrid (SUMMA 112). España

^7^Departamento de Medicina Interna. Hospital Ramón y Cajal. España

^8^Centro Colaborador de la OMS para la Salud Mundial de la Mujer, Instituto de Investigación del Metabolismo y Sistemas. Universidad de Birmingham. REINO UNIDO.

^&^ Blanca Fuentes y Javier Zamora de forma conjunta supervisaron este trabajo y comparten la posición de último autor

^^^ Miembros de la Red Ictus de la Comunidad de Madrid (incluidos en Supporting Information 1).

*Autor de correspondencia:

Nicolás Riera-López: [nicolas.riera@salud.madrid.org](mailto:nicolas.riera@salud.madrid.org)

ORCID: 0000-0002-0862-434X

## ABSTRACT

**Antecedentes**: Son muy pocos los estudios que han investigado el efecto de la pandemia por COVID-19 en el protocolo de código ictus prehospitalario. Durante la primera oleada, España fue uno de los países más afectados por la pandemia de la enfermedad por coronavirus SARS-CoV-2. Esta catástrofe sanitaria eclipsó otras patologías, como el ictus agudo, primera causa de muerte entre las mujeres y primera causa de discapacidad entre los adultos. Cualquier interferencia en el protocolo del código del ictus puede retrasar la administración del tratamiento de reperfusión para los ictus isquémicos agudos, lo que conlleva un peor pronóstico del paciente. Nuestro objetivo es comparar el funcionamiento del código de ictus durante la primera ola de la pandemia en comparación con el mismo periodo del año anterior.

**Métodos**: Estudio observacional multicéntrico de series temporales interrumpidas de la cohorte de códigos de ictus del SUMMA 112 y de los diez hospitales con unidad de ictus de la Comunidad de Madrid. Se establecieron dos grupos en función de la fecha en la que fueron atendidos: el primero durante las fechas de mayor incidencia diaria acumulada de la primera oleada del COVID-19 (del 27 de febrero al 15 de junio de 2020), y el segundo, el mismo periodo del año anterior (del 27 de febrero al 15 de junio de 2019). Para evaluar el rendimiento del código de ictus, se comparó cada uno de los periodos de tiempo del servicio de urgencias prehospitalario, la precisión diagnóstica (proporción de códigos de ictus con un diagnóstico final de ictus agudo sobre el total), la proporción de pacientes tratados con terapias de reperfusión y la mortalidad intrahospitalaria.

**Resultados**: El SUMMA 112 activó el código de ictus en 966 pacientes (514 en el grupo prepandémico y 452 pandémico). El tiempo de gestión de la llamada aumentó un 9% (IC 95%: -0,11; 0,91; valor p = 0,02), y el tiempo en el lugar aumentó un 12% (IC 95%: 2,49; 5,93; valor p = <0,01). La precisión diagnóstica y la proporción de pacientes tratados con terapias de reperfusión permanecieron estables. La mortalidad intrahospitalaria disminuyó en un 4% (p=0,05).

**Conclusiones**: Durante la primera oleada se observó una prolongación del tiempo "en la escena" de la gestión de las llamadas del 112, y del ingreso hospitalario. La precisión diagnóstica prehospitalaria y la proporción de pacientes tratados a nivel hospitalario con trombólisis intravenosa o trombectomía mecánica no se alteraron con respecto al año anterior, lo que demuestra la capacidad de recuperación de la red de ictus y del servicio de emergencias médicas.

## INTRODUCCIÓN

España fue durante la primera ola, uno de los epicentros a nivel mundial de la pandemia por la enfermedad debida al coronavirus SARS-CoV-2 (COVID-19). Hasta el 15 de junio de 2020 en la Comunidad de Madrid se notificaron 70.554 casos y 9.157 fallecidos (1), aunque probablemente ambas cifras infraestimen lo ocurrido, debido a la baja cantidad de pruebas de detección del SARS-CoV-2 que se realizaron en ese momento inicial de la pandemia (2,3). Esta catástrofe sanitaria ha eclipsado al resto de las patologías, produciendo una interferencia considerable en los sistemas sanitarios.

El ictus agudo (IA) es la segunda causa de mortalidad global y la primera de discapacidad en el adulto (4). Constituye la patología tiempo-dependiente que más se atiende en el Servicio de Urgencias Médicas de Madrid (SUMMA 112) (5). Los tratamientos desarrollados durante las dos últimas décadas han permitido mejorar la mortalidad y el grado de dependencia que sufren los pacientes con ictus isquémico agudo, pero los resultados dependen del tiempo que transcurre desde el inicio de los síntomas a la administración del tratamiento de reperfusión. Se ha estimado que cada minuto que se ahorra supone cerca de una semana de vida adicional sin secuelas (6).

La detección de llamadas con síntomas sospechosos de IA, el envío de una ambulancia con la mayor prioridad, la valoración in-situ del paciente, la selección del hospital idóneo alertando al neurólogo de guardia y el traslado urgente, constituyen el núcleo del protocolo de “código ictus” (CI). Su implantación ha supuesto una importante reducción de los tiempos, con la consiguiente influencia en el pronóstico del paciente (7–9). Por ello las guías internacionales recomiendan el acceso al sistema sanitario a través del servicio de emergencias médicas (SEM) mediante la llamada al 112 (10,11). Cualquier interferencia en su funcionamiento podría provocar un retraso en la atención, un retraso en la administración del tratamiento de reperfusión y por ello, un empeoramiento del pronóstico de los pacientes.

Durante la primera ola de la pandemia, varios grupos alertaron sobre una drástica reducción de los casos de ictus agudo (IA), saturación de las centrales de llamadas, retraso en las ambulancias e incluso de saturación en los servicios de urgencias hospitalarios (12–15). Sin embargo, no hay información suficiente sobre los aspectos concretos que se han visto más afectados en la fase prehospitalaria y hospitalaria de la atención urgente al IA. De acuerdo con lo recomendado por investigadores y organismos internacionales, es necesario un examen riguroso de lo que ha ocurrido durante la pandemia por COVID-19 para emitir recomendaciones que permitan mejorar la estabilidad del protocolo en momentos de crisis (16,17).

El objetivo principal del estudio es comparar los períodos de tiempo invertidos en cada una de las fases del CI en la Comunidad de Madrid antes de la irrupción de la pandemia y durante el período con mayor incidencia de la primera ola, tanto en la fase prehospitalaria como en la fase hospitalaria. El objetivo secundario es comparar el resto de aspectos fundamentales del funcionamiento del CI entre los mismos períodos (entre los que se encuentra la concordancia diagnóstica del SEM, la proporción de pacientes tratados, su gravedad o mortalidad).

## MATERIAL Y MÉTODOS

Estudio cuasiexperimental antes-después multicéntrico de la cohorte de CI del SUMMA 112 y de los diez hospitales con Unidad de Ictus de la Comunidad de Madrid. El protocolo se aprobó por el Comité de Ética con Medicamentos de la Comunidad de Madrid (acta 12/2020) que autorizó la exención de consentimiento informado. Los datos anonimizados y agregados están disponibles mediante solicitud razonada.

### Población y entorno

El SUMMA 112 es el principal SEM en la provincia de Madrid (6.5 millones de habitantes en 2020). Anualmente activa el protocolo de CI en cerca de 2,000 pacientes que cumplen los siguientes criterios: síntomas sugestivos de IA, menos de 24 horas de evolución o inicio desconocido, y ausencia de dependencia funcional previa importante. Si se cumplen los criterios, realiza las siguientes acciones:

1. Envía un equipo de emergencias que valora al paciente in-situ.
2. Selecciona el hospital de destino utilizando la escala Madrid-DIRECT (EMD), que detecta a los pacientes que se pueden beneficiar de un tratamiento con trombectomía mecánica (18) (Anexo 1).
3. Finalmente, alerta al neurólogo de guardia y realiza el traslado urgente al hospital idóneo más cercano.

La Red Ictus de la Comunidad de Madrid se compone de hospitales clasificados en niveles en función de su capacidad para atender a los pacientes con IA. Diez de ellos tienen Unidad de Ictus (UI) y capacidad para suministrar tratamiento fibrinolítico, y siete de ellos además tienen capacidad para realizar el tratamiento con trombectomía mecánica en horario laborable, turnándose en grupos de tres fuera de este horario (19).

El primer caso de COVID-19 en la Comunidad de Madrid se notificó el 25 de febrero de 2020. Debido a que el objetivo es analizar lo ocurrido en los momentos de mayor incidencia, se han seleccionado los días que se notificaron incidencias superiores a la mediana de la incidencia acumulada diaria durante la primera ola (1).

De acuerdo con lo anterior, se incluyeron de forma consecutiva a todos los pacientes en los que se activó el CI agrupándolos según la fecha de la llamada al SUMMA 112 en:

1. Grupo pandemia: del 27 de febrero al 15 de junio de 2020.
2. Grupo prepandemia: del 27 de febrero al 15 de junio de 2019, el mismo período del año anterior.

### Recogida de datos

Se enlazaron la base de datos prospectiva de CI del SUMMA 112 con el Conjunto Mínimo Básico de Datos (CMBD) al alta hospitalaria de los diez hospitales con Unidad de Ictus (20).

Para asegurar que la información correspondía al mismo paciente, ambas bases de datos fueron encadenadas mediante un campo cifrado unidireccionalmente del Código de Identificación Personal Autonómico del Sistema Nacional de Salud (CIPA) (21).

### Variables analizadas

Para el objetivo principal se analizaron los períodos de tiempo invertidos en cada uno de los pasos de la atención prehospitalaria y hospitalaria.

Para el objetivo secundario, se recogieron variables clínicas, de proceso y de resultado de la fase prehospitalaria y hospitalaria del CI, extraídas de las recomendaciones internacionales de y utilizadas en registros de control de calidad (10,11,22).

En la fase prehospitalaria se analizaron la edad, sexo, constantes vitales recogidas por el SUMMA 112 (tensión arterial, frecuencia cardíaca, frecuencia respiratoria, saturación de oxígeno, glucemia, temperatura y ritmo electrocardiográfico), sospecha de oclusión de gran vaso (medida con la Escala Madrid Direct) y área de ingreso hospitalaria.

Y en la fase hospitalaria se analizaron la concordancia entre la sospecha clínica prehospitalaria y el diagnóstico al alta hospitalaria (medida como porcentaje de diagnósticos en los que el SEM y hospital coinciden en IA, sin distinguir entre ictus isquémico o hemorrágico), el porcentaje de ingreso en unidad de cuidados intensivos (UCI), de tratamientos de reperfusión (fibrinolisis y trombectomía mecánica) sobre el total de pacientes. Se analizó también la gravedad de los pacientes utilizando tres parámetros: el índice de comorbilidad de Charlson con pesos actualizados (que evalúa la esperanza de vida a 10 años, clasificando la comorbilidad del paciente en ausente, baja o alta) (23,24), grado de severidad y riesgo de mortalidad (que establecen cuatro grados de riesgo: menor, moderado, mayor o extremo) (25). Y finalmente altas por exitus.

### Análisis estadístico

Las variables categóricas se presentan mediante frecuencias absolutas y relativas, y las variables cuantitativas mediante medias y la desviación estándar en caso de variables normales, o de medianas y rangos intercuartílicos para variables no normales. Las diferencias entre los dos grupos de análisis fueron evaluadas mediante pruebas de t-Student o pruebas de U de Mann-Whitney y pruebas de Chi-Cuadrado según corresponda.

Para evaluar la magnitud de las diferencias, se estimaron los intervalos de confianza para la diferencia de medianas mediante técnicas de bootstrap (800 repeticiones). Las distribuciones de los tiempos en ambos periodos se mostraron gráficamente en forma de box-plots. Para evaluar el efecto directo de la pandemia en los tiempos de intervención, así como para analizar eventuales tendencias temporales, se llevó a cabo un análisis de series temporales interrumpidas mediante modelos de regresión lineal. Se reportan las pendientes de los periodos prepandemia y pandemia y el efecto de la pandemia a 27 de febrero de 2020 con sus respectivos intervalos de confianza al 95 %. Para los análisis se utilizó el software estadístico Stata v16 (Statacorp LLC, College Station, Texas).

## RESULTADOS

Durante la primera ola de la pandemia, la mediana de incidencia acumulada diaria fue 98 casos de COVID-19 por cada 100.000 habitantes, detectada por test de antígenos. Las fechas con incidencias superiores definieron al grupo de pandemia. El SUMMA 112 activó el CI en 966 pacientes (514 en el grupo prepandemia y 452 pandemia). En 88 (17%) pacientes en el periodo prepandemia y 83 (18%) en pandemia no se identificó el registro CMBD hospitalario correspondiente. La figura 1 muestra el gráfico de flujo de los pacientes.

Figura 1: Diagrama de flujo de los pacientes


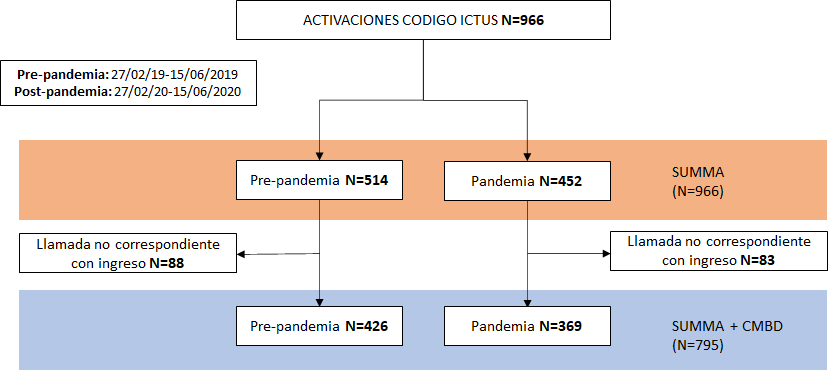


Las características basales de los pacientes atendidos por el SEM se presentan en la tabla 1. Las variables recogidas durante la hospitalización se muestran en la tabla 2. Durante la pandemia se atendieron un 6.4% menos de CI en comparación con el período prepandemia. Los pacientes fueron más jóvenes (70 años vs. 72, diferencia 2.2; 95%IC: 0.29;4.0; p valor= 0.02) y con mayor proporción de sexo masculino (54.8% vs. 47.3%, diferencia 6.5%; 95%IC:0.2;12.78; p valor=0.04).

Tabla 1: Baseline characteristics

| **Variables prehospitalarias** | | N_0_\|N_1_ | Prepandemia | Pandemia | P valor |
| --- | --- | --- | --- | --- | --- |
| Sexo | |  |  |  |  |
| Hombres | | 514\|452 | 243 (47.3%) | 243 (53.8%) | 0.04* |
| Edad (años) | | 514\|452 | 72.4(14.4) | 70.2(15.0) | 0.01** |
| Mas de 80 años | |  | 182(35.4%) | 128 (28.3%) | 0.02* |
| Presión arterial sistólica (mmHg) | | 492\|444 | 155.9(28.7) | 155.3(30.5) | 0.57 |
| Presión arterial diastólica (mmHg) | | 492\|446 | 87.2 (18.9) | 87.7 (20.1) | 0.84 |
| Frecuencia cardíaca (lpm) | | 491\|438 | 84.6 (23.7) | 83.9 (22.2) | 0.79 |
| Frecuencia respiratoria (rpm) | | 237\|310 | 15.8 (4.8) | 15.5 (4.1) | 0.52 |
| Saturación O2 (%) | | 452\|418 | 94.8 (5.0) | 94.8 (4.1) | 0.97 |
| Nivel de glucemia (mg/dl) | | 482\|426 | 134.2 (47.1) | 134.4 (47.1) | 0.81 |
| Temperatura (ºC) | | 425\|392 | 35.95 (0.6) | 35.97 (0.7) | 0.51 |
| Electrocardiograma n(%) | | 470\|375 | 415 (88.3%) | 293 (78.1%) | <0.01** |
| Escala Madrid-DIRECT (n) | | 282\|322 |  |  |  |
| Positivos (EMD>1) | |  | 76 (14.8%) | 104 (23%) | 0.15 |
| Ritmo (n) | | 407\|339 |  |  |  |
| Bloqueo AV | |  | 5 (1.2%) | 13 (3.8%) | 0.13 |
| Fibrilación Auricular | |  | 81 (19.9%) | 59 (17.4%) |  |
| Ritmo de Marcapasos | |  | 15 (3.7%) | 9 (2.7%) |  |
| Sinusal | |  | 304 (74.7%) | 252 (74.3%) |  |
| Otros | |  | 2 (1.1%) | 6 (1.8%) |  |
| Vías periféricas n(%) | | 471\|376 | 422 (89.6%) | 317 (84.3%) | 0.02* |
| Lugar de transferencia (n) | | 228\|259 |  |  |  |
| Box vital | |  | 174 (76.3%) | 223 (86.1%) | 0.01** |
| Otros | |  | 54 (23.7%) | 36 (13.9%) |  |
| Escala de Coma de Glasgow (xx) | | 464\|420 | 13.2 (2,7) | 13.3 (2,6) | 0.96 |
| **Variables hospitalarias** | |  | Prepandemia | Pandemia | P valor |
| Paso UCI n(%) | | 426\|369 | 108 (25.4%) | 63 (17.1%) | <0.01** |
| Número de diagnósticos n(%) | | 426\|369 | 11.8(4.8) | 12.2(4.5) | 0.33 |
| Índice de comorbilidad de Charlson (xx) | | 426\|369 | 1.6(2.6) | 1.8(4.0) | 0.87 |
| Peso grd  (xx) | | 426\|369 | 1.4(1.6) | 1.2(1.1) | 0.66 |
| Tratamiento IA Isquémico n(%) | |  |  |  |  |
| Trombectomías | | 426\|369 | 83 (19.9%) | 69(18.9%) | 0.74 |
| Fibrinolisis | | 426\|369 | 61 (16.7%) | 70(16.8%) | 0.99 |
| Riesgo de mortalidad n(%) | | 426\|369 |  |  |  |
|  | Menor |  | 91 (21.4%) | 83 (22.5%) | 0.05 |
|  | Moderado |  | 207 (48.6%) | 168 (45.5%) |  |
|  | Mayor |  | 70 (16.4%) | 84 (22.8%) |  |
|  | Extremo |  | 58 (13.6%) | 34 (9.2%) |  |
| Riesgo de severidad n(%) | | 426\|369 |  |  |  |
|  | Menor |  | 66 (15.5%) | 54 (14.6%) | 0.06 |
|  | Moderado |  | 172 (40.4%) | 137 (37.1%) |  |
|  | Mayor |  | 129 (30.3%) | 142 (38.5%) |  |
|  | Extremo |  | 59 (13.8%) | 36 (9.8%) |  |
| Exitus n(%) | | 426\|369 | 58(13.6%) | 34 (9.2%) | 0.05 |

N_0:_ Número de pacientes periodo Prepandemia N_1_ Número de pacientes periodo Pandemia

Tabla 2: Diagnósticos al alta hospitalaria

| **Diagnóstico ICTUS** | Prepandemia N=426 | Pandemia N=369 | P valor |
| --- | --- | --- | --- |
| Codigo Ictus |  |  |  |
| No concordante | 48 (11.2%) | 53 (14.4%) |  |
| Concordante SEM - Hospital | 378 (88.7%) | 316 (85.6%) | 0.19 |
| De los no concordantes |  |  |  |
| Infecciones cerebrales, meníngeas | 4 (8%) | 3 (6%) | 0.79 |
| Otras infecciones: Sepsis, neumonía, empiema, absceso retroperitoneal | 4 (8%) | 6 (11%) |  |
| Otros canceres y tumores | 2 (4%) | 3 (6%) |  |
| Tumores cerebrales y meníngeos y metastásicos | 5 (10%) | 7 (13 %) |  |
| Hiponatremia, diabetes | 2 (4%) | 1 (2%) |  |
| Amiloidosis localizada, delirium, disautonomías, Horner, Hipotensión ortostática, quistes | 11 (23%) | 11 (21%) |  |
| Epilepsias y convulsiones | 10 (21%) | 17 (32%) |  |
| Migrañas y cefaleas | 2 (4%) | 1 (2%) |  |
| IAM, TEP, valvulopatías, arritmias, EPOC, asma, atelectasia, insuficiencia hepática, autoinmunes | 8 (17%) | 4 (8%) |  |

La figura 2 describe cada uno de los períodos de tiempo del proceso asistencial urgente. Durante la pandemia todos ellos aumentaron en mayor o menor medida (tabla 3 y figura 3). Entre los tiempos de intervención prehospitalarios destaca el tiempo de la gestión de la llamada por parte del centro coordinador (paso 1) que aumentó casi medio minuto (4.8 minutos vs. 4.4; diferencia 0,4; 95% IC: -0.11;0.91; p.valor=0.02), y el tiempo en escena (paso 4) que aumentó un 12% (37.6 minutos vs. 33.5, diferencia 4.1; 95% IC: 2.49; 5.93; p.valor=<0.01). En cuanto a los tiempos hospitalarios, el periodo desde la llegada del paciente al hospital y el momento del ingreso (paso 6) se incrementó un 10% (2.5 horas vs. 2.3, diferencia 0.2; 95% IC: -0.23;0.87; p-valor=0.02). En la serie temporal interrumpida se observa el cambio directo producido por el efecto de la pandemia en el tiempo “en escena”, y aunque con una pendiente no significativa (pendiente -0.01 95% IC: -0.04; 0.02) se fue reduciendo con el paso del tiempo (figura 4).

Figura 2: Diagrama de flujo de los períodos de tiempo analizados


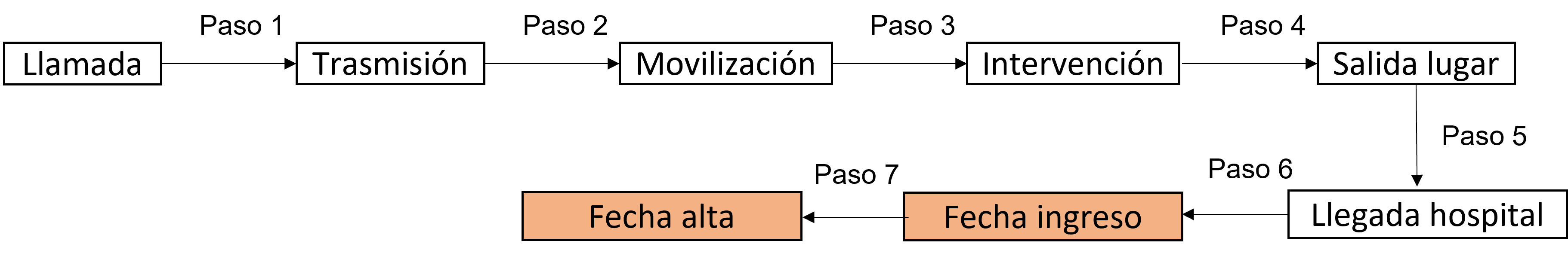


Figura 3: Gráfico de cajas con los tiempos prehospitalarios en cada una de las fases de la atención sanitaria.

a. La llamada es recogida por el operador del 112.

b. Se envía un mensaje con los datos de la misión a la ambulancia.

c. La ambulancia comienza a moverse hacia la dirección de la misión.

d. La ambulancia se detiene al llegar a la dirección de la misión.

e. La ambulancia con el paciente comienza a moverse hacia el hospital.

f. La ambulancia con el paciente se detiene al llegar al hospital.

g. El paciente es dado de alta del hospital (a su casa o a otro centro de salud).


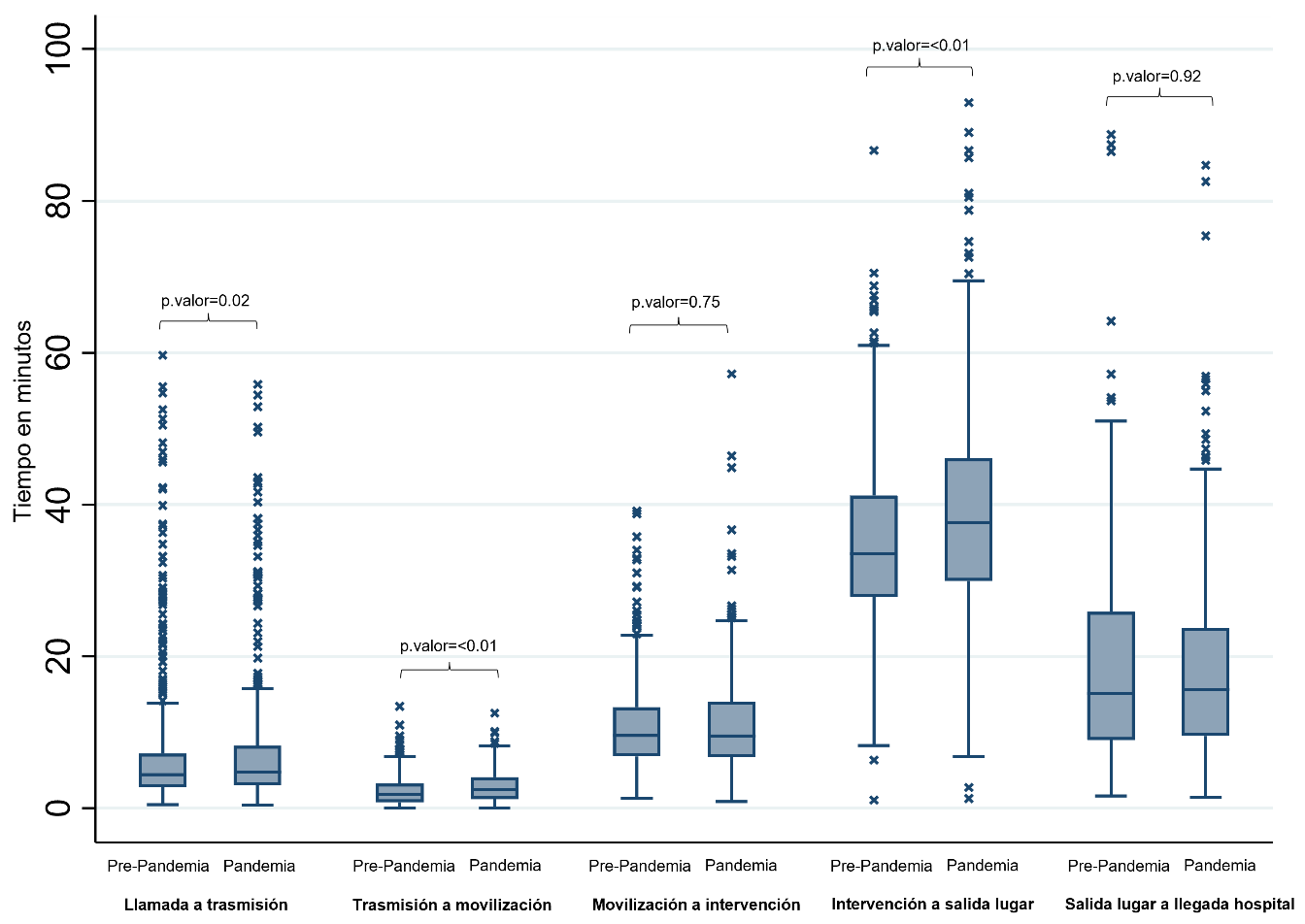


Figura 4: Serie temporal interrumpida con el tiempo “en escena” del SUMMA 112 (desde que llega al lado del paciente, hasta que sale hacia el hospital)


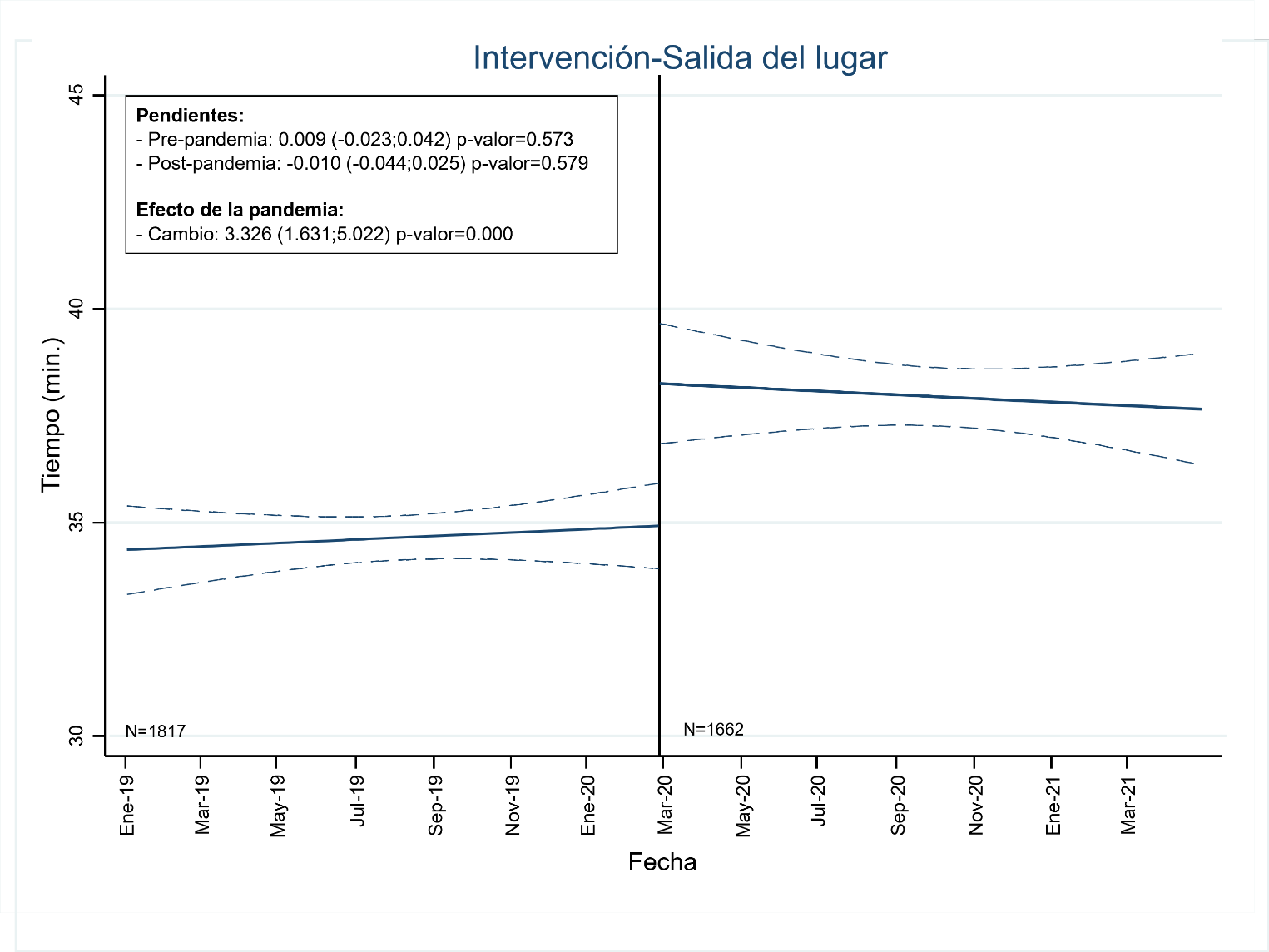


Tabla 3: Tiempos invertidos en cada una de las fases de atención prehospitalaria y hospitalaria

| **Tiempos extra-hospitalarios (minutos)** | | | | | |
| --- | --- | --- | --- | --- | --- |
|  | Pre-pandemia | | Pandemia | |  |
|  | N | Mediana(IQR) | N | Mediana(IQR) | *P valor |
| Llamada-Trasmisión | 506 | 4.4 (2.8;7.2) | 436 | 4.8 (3.0;8.2) | 0.02 |
| Trasmisión-Movilización | 506 | 1.9 (0.8;3.2) | 441 | 2.4 (1.2;4.1) | <0.01 |
| Movilización-Intervención | 492 | 9.6 (6.8;13.3) | 427 | 9.5 (6.9;14.0) | 0.75 |
| Intervención-Salida lugar | 478 | 33.5 (27.9;41.2) | 407 | 37.6 (29.9;46.1) | <0.01 |
| Salida-lugar llegada al hospital | 439 | 15.2 (9.0;25.9) | 372 | 15.6 (9.5;23.8) | 0.92 |
| Llamada-Llegada al hospital | 449 | 72.3 (59.9;86.6) | 383 | 75.4 (64.0;91.37) | <0.01 |
| **Tiempos intra-hospitalarios (horas)** | | | | | |
|  | Pre-pandemia | | Pandemia | | *P valor |
| Llegada hospital- ingreso | 378 | 2.3 (1.2;4.9) | 323 | 2.5 (1.3;7.9) | 0.02 |
| Ingreso hospital- Alta hospital | 426 | 138.1 (65.5;266.7) | 369 | 142.4 (73.4;289.9) | <0.01 |
| Llega hospital-Alta | 378 | 145.0 (69.6;279.5) | 323 | 148.5 (77.0;297.2) | <0.01 |

Ni el diagnóstico concordante del SUMMA 112 con el alta hospitalaria, ni el análisis de las discrepancias diagnósticas por patología CMBD presentó diferencias significativas entre los dos períodos.

Los resultados sobre el recorrido hospitalario de los pacientes mostraron diferencias en el paso en por UCI que se redujo un 8%. No se encontraron diferencias significativas en el porcentaje de pacientes tratados con fibrinolítico intravenoso o trombectomía mecánica.

No se detectaron diferencias estadísticamente significativas en las constantes vitales recogidas, en el Índice de Comorbilidad de Charlson, en el riesgo de mortalidad ni en el de severidad. La EMD no mostró diferencias significativas en la proporción de pacientes con puntuación positiva. Se redujo la proporción de pacientes a los que se le hizo un electrocardiograma casi un 10%. La proporción de altas por exitus pasó de un 13% a un 9%.

## DISCUSIÓN

Mientras que los tiempos de tránsito de las ambulancias (pasos 3 y 5) no sufrieron variaciones significativas (pese a que las medidas de confinamiento influyeron positivamente en el tráfico durante la primera ola), los tiempos de respuesta de la central de llamadas del 112 (paso 1) y los tiempos en escena (paso 4) aumentaron más de un 10% durante la primera ola de la pandemia (figura 3). Todos los tiempos hospitalarios aumentaron en torno a un 10%.

Las guías de práctica clínica sobre IA recomiendan tiempos de respuesta del centro de llamadas inferior a 1 minuto y en escena inferiores a 15 minutos (26). El correcto funcionamiento de la central de llamadas es esencial para el manejo urgente de patologías que pueden poner en peligro la vida (27). La avalancha de llamadas de pacientes con síntomas de COVID-19 pudo saturar en ciertos momentos el centro de recepción de llamadas del SUMMA-112, causando una gran interferencia en la atención del resto de llamadas urgentes. Durante los peores días de la primera ola, se llegaron a triplicar las llamadas que se recibían en el mismo período el año anterior. En la zona de Lausanne (Suiza) aumentaron un 212%, en París un 225%, en Emilia Romana (Italia) se duplicaron y en Cataluña (España) se triplicaron (12,28). Hasta que no se estableció por las autoridades sanitarias un número alternativo para las consultas relacionadas con el COVID-19, muchos de los pacientes utilizaron el 112 para resolver sus dudas y pedir asistencia. Por ello, a nuestro juicio, es muy importante establecer métodos de control de desbordamiento de llamadas, para evitar que en situaciones similares las esperas para ser atendidos se prolonguen excesivamente. Utilizar una aplicación de autodiagnóstico, o derivar la avalancha de llamadas de pacientes con dudas sobre la COVID-19 a otro número que no sea el de emergencias, son potenciales soluciones que han demostrado su efectividad (29).

Con respecto al tiempo en escena (paso 4), el cambio más relevante fue el uso de los equipos de protección individual (EPI) contra riesgos biológicos que debían colocarse justo antes de atender al paciente. El proceso de colocación debe ser ordenado, secuencial y supervisado por otro miembro del equipo para evitar errores. Y en los primeros días la falta de destreza en el procedimiento pudo provocar el aumento del tiempo registrado. Un estudio en el Oeste de Pensilvania, implicando a 22 agencias de emergencias (urbanas, suburbanas y rurales), mostró un aumento de cercano al 10% (15.7±9.5 a 18.3±10.8) para todas las patologías (30). Incluso en regiones mínimamente afectadas como Okayama, con solo 16 casos de COVID-19 durante la primera oleada, se produjo un incremento similar al nuestro del tiempo en escena, por lo que creemos que es un efecto independiente de la incidencia de COVID-19 (31). En el análisis de la serie temporal interrumpida se observó una reducción progresiva del mismo (figura 4). Creemos que según el entrenamiento producía sus efectos, los equipos fueron capaces de reducir el tiempo en escena. El entrenamiento en estas habilidades no solo reduce el tiempo que tardan los equipos en la colocación y retirada del EPI, sino la confianza en que el proceso se está haciendo con la seguridad requerida.

El tiempo hasta el ingreso del paciente también aumentó, circunstancia que incide negativamente en el pronóstico del paciente, no ya por el eventual retraso en la administración del tratamiento de reperfusión, o por el retraso en los cuidados especializados que estos pacientes reciben en las Unidades de Ictus, sino de forma independiente (32–34). Otros grupos han informado de un importante incremento de la mediana de la estancia hospitalaria de hasta 8 días (35), mientras que en nuestra serie no llega a las 3 horas. Una de las principales razones para no prolongar la estancia hospitalaria es la necesidad de liberar camas para poder ingresar a pacientes de urgencias. La situación de saturación hospitalaria creemos que retrasó todos los procesos, pero no en gran medida en nuestra serie.

Nuestro SEM mantuvo un porcentaje superior al 85% de concordancia diagnóstica que no sufrió variaciones significativas durante la pandemia. El aumento del número de traslados de pacientes con stroke mimics habría supuesto una importante sobrecarga al sistema ya diezmado por la propia pandemia. Los SEM de otros países tienen una proporción más baja (36–38). La fortaleza de nuestro protocolo reside en el despacho de estos avisos a equipos con personal sanitario de alta cualificación (médicos y enfermeras), que son capaces de distinguir con mayor precisión los síntomas neurológicos que corresponden al ictus agudo, como ocurre en otros países con SEM similares (39).

El circuito que los pacientes recorrieron en el hospital también se vio afectado, con una menor proporción de ellos ingresando directa o diferidamente en las UCI. La saturación de las UCI con pacientes COVID-19 podría explicar este hallazgo.

En cuanto a los tratamientos del IA isquémico, pese a la saturación del sistema sanitario durante la primera ola, la Red Ictus de la Comunidad de Madrid ha sido capaz de mantener la misma proporción de fibrinolisis y trombectomías mecánicas. Muchos de los estudios analizados reflejan un menor porcentaje de tratamientos, más llamativo en centros con un alto volumen de ingresos por COVID-19 (40). Un estudio francés informaba de un descenso de más del 20% de las trombectomías, con un aumento de más de un 10% de los tiempos puerta-ingle (41). Esto demuestra una gran resiliencia de nuestra red ya que, en circunstancias de muy alta presión asistencial, ha sido capaz de mantener proporciones similares de pacientes tratados.

No encontramos una explicación sencilla para la reducción de la frecuencia de mortalidad hospitalaria en pacientes con ictus durante la primera oleada de la pandemia. No hay datos en la estimación de gravedad de ambos grupos que lo justifiquen (ni en los índices de Charlson, ni en el resto de índices analizados). Una posibilidad, concordante con el hallazgo de una menor media de edad durante este período, es que muchos pacientes ancianos no buscasen asistencia ante la presencia de síntomas de ictus, o que los mismos no fueran trasladados a los hospitales ante la saturación de las urgencias. También es posible que muchos de los fallecidos por ictus, al tener infección concomitante por COVID-19, ésta se haya considerado como causa del exitus y no el ictus.

La utilización los datos obtenidos por el SUMMA 112 encadenados con los datos administrativos hospitalarios extraídos del informe de alta de los pacientes usando el CMBD, nos ha permitido obtener información sobre los aspectos de la atención urgente del IA en la Comunidad de Madrid que más se han visto afectados durante la primera ola de la pandemia, así como su efecto en el ámbito terapéutico y pronóstico.

Nuestro estudio presenta tres limitaciones fundamentales. La primera es que se refiere únicamente a los ictus atendidos por el SUMMA 112, no teniendo en cuenta a los que ingresan en el hospital por otros medios. Sin embargo, es importante destacar que habitualmente se trata de los pacientes con ictus más graves y, por tanto, los que más se verían afectados por un desajuste en el protocolo CI (42,43). La segunda es que los datos hospitalarios no han podido recogerse en la totalidad de los pacientes. La mayor parte de registros perdidos en el CMBD se debió a la ausencia del CIPA, fundamentalmente en el caso de pacientes que proceden de otras comunidades autónomas, de aseguradoras privadas, o porque dicho dato no pudo ser recogido por el SUMMA 112. En estos casos solo se analizaron las variables prehospitalarias sin poder analizar las hospitalarias. No obstante, esto afectó a una pequeña proporción de nuestra población y pensamos que no resta fortaleza a los resultados obtenidos. Y la tercera, la precisión del CMBD para diagnósticos y tratamientos. Hay estudios que han cifrado su sensibilidad para ictus agudo superior al 82% y su especificidad superior al 95% (44). No hemos encontrado literatura que analice la validez con respecto a los tratamientos y procedimientos terapéuticos. Pero creemos que el uso del CMBD es una alternativa cuando los registros que rellenan manualmente los profesionales están incompletos debido a la sobrecarga de trabajo que ha acarreado la pandemia.

## CONCLUSIONES

La primera ola de pandemia definitivamente incidió en importantes aspectos del funcionamiento del CI en nuestro medio. Se incrementaron cerca de un 10% los tiempos de atención telefónica, en escena, el tiempo hasta el ingreso y estancia hospitalaria. La precisión diagnóstica de los profesionales del SEM no se vio afectada de forma significativa, mostrando una de las fortalezas que tienen los equipos sanitarios del SUMMA 112. Finalmente, tampoco la proporción de paciente tratados con fibrinolisis o trombectomía mecánica no se ha reducido durante la primera ola de la pandemia, demostrando la gran resiliencia de la red de ictus de la Comunidad de Madrid.

## Agradecimientos

A María José Medrano y Gonzalo Bayo del Departamento de Informática del SUMMA 112 por su desarrollo informático para la recogida de las variables. A la mesa de coordinación de enfermería. Al Profesor Exuperio Díez Tejedor (Jefe de Servicio de Neurología del Hospital Universitario de La Paz), al Dr. José Egido Herrero (Jefe de Servicio de Neurología del Hospital Universitario Clínico San Carlos), Dr. Joaquín Carneado (UI Hospital Puerta de Hierro Majadahonda), Dra. Patricia Calleja Castaño (Jefe de Sección de la UI del Hospital Universitario Doce de Octubre), Dr. Jaime Masjuán Vallejo (Jefe de Servicio de Neurología del Hospital Universitario Ramón y Cajal), Dr. Antonio Gil Núñez (Jefe de Servicio de Neurología del Hospital Universitario Gregorio Marañón), Dr. José Vivancos Mora (Jefe de Servicio de Neurología del Hospital Universitario de la Princesa), miembros del grupo multidisciplinar del Pan Ictus de la Comunidad de Madrid. A todos los profesionales del SUMMA 112 y hospitalarios de la Red Ictus de la Comunidad de Madrid que en estas extraordinarias circunstancias han sabido mantener un compromiso con sus pacientes y con la institución sanitaria a nuestro juicio heroica.

## FINANCIACIÓN

Financiado por la Fundación para la Investigación e Innovación Biosanitaria en Atención Primaria (FIIBAP) y la Consejería de Sanidad de la Comunidad de Madrid a través de subvenciones a fondo perdido de los créditos concedidos a la Comunidad de Madrid por el Fondo COVID-19 del Gobierno de España, recogido en la Orden HAC/667/2020.

## ABREVIATURAS

CIPA Código de Identificación Personal Autonómico del Sistema Nacional de Salud

COVID-19 Enfermedad por el nuevo coronavirus 2019

SARS_CoV-2 Coronavirus del síndrome respiratorio agudo grave 2

IA Ictus Agudo

SUMMA-112 Servicio de Urgencias Médicas de Madrid 112

CI Código Ictus

SEM Servicio de Emergencias Médicas

EMD Escala Madrid Direct

CIPA Código de Identificación de Paciente Autonómico

UI Unidad de Ictus

## CONFLICTO DE INTERESES

Nicolás Riera-López y Jorge Rodríguez-Pardo de Donlebún, han recibido pagos por cursos de formación de la Iniciativa Angels (Boheringer Ingelheim). El resto de los autores no informan de ningún conflicto de interés.

## REFERENCIAS

1. Centro Nacional de Epidemiología. Instituto de Salud Carlos III. Ministerio de Sanidad. Gobierno de España. COVID-19. Documentación y datos [Internet]. [citado 9 de febrero de 2022]. Disponible en: https://cnecovid.isciii.es/covid19/#documentaci%C3%B3n-y-datos

2. Pollán M, Pérez-Gómez B, Pastor-Barriuso R, Oteo J, Hernán MA, Pérez-Olmeda M, et al. Prevalence of SARS-CoV-2 in Spain (ENE-COVID): a nationwide, population-based seroepidemiological study. Lancet. 22 de agosto de 2020;396(10250):535-44.

3. León-Gómez I, Mazagatos C, Delgado-Sanz C, Frías L, Vega-Piris L, Rojas-Benedicto A, et al. The Impact of COVID-19 on Mortality in Spain: Monitoring Excess Mortality (MoMo) and the Surveillance of Confirmed COVID-19 Deaths. Viruses. 3 de diciembre de 2021;13(12):2423.

4. Ding Q, Liu S, Yao Y, Liu H, Cai T, Han L. Global, Regional, and National Burden of Ischemic Stroke, 1990-2019. Neurology. 18 de enero de 2022;98(3):e279-90.

5. Memorias SUMMA 112 [Internet]. SUMMA 112. 2019 [citado 10 de febrero de 2022]. Disponible en: https://www.comunidad.madrid/hospital/summa112/nosotros/memorias-summa-112

6. Meretoja A, Keshtkaran M, Tatlisumak T, Donnan GA, Churilov L. Endovascular therapy for ischemic stroke: Save a minute-save a week. Neurology. 30 de mayo de 2017;88(22):2123-7.

7. Belvís R, Cocho D, Martí-Fàbregas J, Pagonabarraga J, Aleu A, García-Bargo MD, et al. Benefits of a Prehospital Stroke Code System. Cerebrovasc Dis. 2005;19(2):96-101.

8. Alonso de Leciñana-Cases M, Gil-Núñez A, Díez-Tejedor E. Relevance of Stroke Code, Stroke Unit and Stroke Networks in Organization of Acute Stroke Care – The Madrid Acute Stroke Care Program. Cerebrovasc Dis. 2009;27(1):140-7.

9. Chen C-H, Tang S-C, Tsai L-K, Hsieh M-J, Yeh S-J, Huang K-Y, et al. Stroke Code Improves Intravenous Thrombolysis Administration in Acute Ischemic Stroke. Minnerup J, editor. PLoS ONE. 11 de agosto de 2014;9(8):e104862.

10. Powers WJ, Rabinstein AA, Ackerson T, Adeoye OM, Bambakidis NC, Becker K, et al. Guidelines for the Early Management of Patients With Acute Ischemic Stroke: 2019 Update to the 2018 Guidelines for the Early Management of Acute Ischemic Stroke: A Guideline for Healthcare Professionals From the American Heart Association/American Stroke Association. Stroke. diciembre de 2019;50(12):e344-418.

11. Kobayashi A, Czlonkowska A, Ford GA, Fonseca AC, Luijckx GJ, Korv J, et al. European Academy of Neurology and European Stroke Organization consensus statement and practical guidance for pre‐hospital management of stroke. Eur J Neurol. marzo de 2018;25(3):425-33.

12. Montaner J, Barragán-Prieto A, Pérez-Sánchez S, Escudero-Martínez I, Moniche F, Sánchez-Miura JA, et al. Break in the Stroke Chain of Survival due to COVID-19. Stroke. agosto de 2020;51(8):2307-14.

13. Holmes JL, Brake S, Docherty M, Lilford R, Watson S. Emergency ambulance services for heart attack and stroke during UK’s COVID-19 lockdown. Lancet. 23 de mayo de 2020;395(10237):e93-4.

14. Aguiar de Sousa D, Sandset EC, Elkind MSV. The Curious Case of the Missing Strokes During the COVID-19 Pandemic. Stroke. julio de 2020;51(7):1921-3.

15. Riera-López N, Fuentes B, de Donlebún JR-P. Effect of the COVID-19 pandemic in stroke code activations in the region of Madrid: A retrospective study. Medicine (Baltimore). 29 de octubre de 2021;100(43):e27634.

16. García-Basteiro A, Alvarez-Dardet C, Arenas A, Bengoa R, Borrell C, Del Val M, et al. The need for an independent evaluation of the COVID-19 response in Spain. Lancet. 22 de agosto de 2020;396(10250):529-30.

17. Legido-Quigley H, Mateos-García JT, Campos VR, Gea-Sánchez M, Muntaner C, McKee M. The resilience of the Spanish health system against the COVID-19 pandemic. The Lancet Public Health. mayo de 2020;5(5):e251-2.

18. Rodríguez-Pardo J, Riera-López N, Fuentes B, Alonso de Leciñana M, Secades-García S, Álvarez-Fraga J, et al. Prehospital selection of thrombectomy candidates beyond large vessel occlusion: M-DIRECT scale. Neurology. 25 de febrero de 2020;94(8):e851-60.

19. Pastor S, de Celis E, Losantos García I, Alonso de Leciñana M, Fuentes B, Díez-Tejedor E, et al. Development of the Madrid Stroke Programme: Milestones and Changes in Stroke Trends and Mortality from 1997 to 2017. Neuroepidemiology. 2021;55(2):135-40.

20. Gonzalo SE. Explotación estadística del Conjunto Mínimo Básico de Datos Hospitalarios. :4.

21. Agencia Española de Protección de Datos. Introducción al HASH como técnica de seudoanonimización de datos personales [Internet]. [citado 1 de febrero de 2022]. Disponible en: https://www.aepd.es/sites/default/files/2020-05/estudio-hash-anonimidad.pdf

22. Mikulik R, Bar M, Grecu A, Herzig R, Neumann J, Sanak D, et al. The registry of stroke care quality (RES-Q): The first nation-wide data on stroke care quality. Journal of the Neurological Sciences. octubre de 2017;381:91.

23. Deyo RA, Cherkin DC, Ciol MA. Adapting a clinical comorbidity index for use with ICD-9-CM administrative databases. J Clin Epidemiol. junio de 1992;45(6):613-9.

24. Quan H, Li B, Couris CM, Fushimi K, Graham P, Hider P, et al. Updating and validating the Charlson comorbidity index and score for risk adjustment in hospital discharge abstracts using data from 6 countries. Am J Epidemiol. 15 de marzo de 2011;173(6):676-82.

25. Averill RF, Goldfield NI, Muldoon J, Steinbeck BA, Grant TM. A closer look at all-patient refined DRGs. J AHIMA. enero de 2002;73(1):46-50.

26. Jauch EC, Saver JL, Adams HP, Bruno A, Connors JJ (Buddy), Demaerschalk BM, et al. Guidelines for the Early Management of Patients With Acute Ischemic Stroke: A Guideline for Healthcare Professionals From the American Heart Association/American Stroke Association. Stroke. marzo de 2013;44(3):870-947.

27. Kurz MC, Bobrow BJ, Buckingham J, Cabanas JG, Eisenberg M, Fromm P, et al. Telecommunicator Cardiopulmonary Resuscitation: A Policy Statement From the American Heart Association. Circulation. 24 de marzo de 2020;141(12):e686-700.

28. Al Amiry A, Maguire BJ. Emergency Medical Services (EMS) Calls During COVID-19: Early Lessons Learned for Systems Planning (A Narrative Review). OAEM. septiembre de 2021;Volume 13:407-14.

29. Jensen T, Holgersen MG, Jespersen MS, Blomberg SN, Folke F, Lippert F, et al. Strategies to Handle Increased Demand in the COVID-19 Crisis: A Coronavirus EMS Support Track and a Web-Based Self-Triage System. Prehospital Emergency Care. 2 de enero de 2021;25(1):28-38.

30. Satty T, Ramgopal S, Elmer J, Mosesso VN, Martin-Gill C. EMS responses and non-transports during the COVID-19 pandemic. The American Journal of Emergency Medicine. abril de 2021;42:1-8.

31. Ageta K, Naito H, Yorifuji T, Obara T, Nojima T, Yamada T, et al. Delay in Emergency Medical Service Transportation Responsiveness during the COVID-19 Pandemic in a Minimally Affected Region [Internet]. Okayama University Medical School; 2020 [citado 16 de febrero de 2022]. Disponible en: https://doi.org/10.18926/AMO/61210

32. Darehed D, Blom M, Glader E-L, Niklasson J, Norrving B, Eriksson M. In-Hospital Delays in Stroke Thrombolysis: Every Minute Counts. Stroke. agosto de 2020;51(8):2536-9.

33. Boulain T, Malet A, Maitre O. Association between long boarding time in the emergency department and hospital mortality: a single-center propensity score-based analysis. Intern Emerg Med. abril de 2020;15(3):479-89.

34. Jones S, Moulton C, Swift S, Molyneux P, Black S, Mason N, et al. Association between delays to patient admission from the emergency department and all-cause 30-day mortality. Emerg Med J. 18 de enero de 2022;emermed-2021-211572.

35. Arsovska A, Mikulik R, Bornstein N. Impact of COVID-19 pandemic on acute stroke care-data analysis from the RES-Q registry 2020 in N. Macedonia. Journal of the Neurological Sciences. octubre de 2021;429:119866.

36. Neves Briard J, Zewude RT, Kate MP, Rowe BH, Buck B, Butcher K, et al. Stroke Mimics Transported by Emergency Medical Services to a Comprehensive Stroke Center: The Magnitude of the Problem. J Stroke Cerebrovasc Dis. octubre de 2018;27(10):2738-45.

37. Hemmen TM, Meyer BC, McClean TL, Lyden PD. Identification of Nonischemic Stroke Mimics Among 411 Code Strokes at the University of California, San Diego, Stroke Center. Journal of Stroke and Cerebrovascular Diseases. enero de 2008;17(1):23-5.

38. H. Buck B, Akhtar N, Alrohimi A, Khan K, Shuaib A. Stroke mimics: incidence, aetiology, clinical features and treatment. Annals of Medicine. 1 de enero de 2021;53(1):420-36.

39. Schewe J-C, Kappler J, Dovermann K, Graeff I, Ehrentraut SF, Heister U, et al. Diagnostic accuracy of physician-staffed emergency medical teams: a retrospective observational cohort study of prehospital versus hospital diagnosis in a 10-year interval. Scand J Trauma Resusc Emerg Med. diciembre de 2019;27(1):36.

40. Nogueira RG, Abdalkader M, Qureshi MM, Frankel MR, Mansour OY, Yamagami H, et al. Global impact of COVID-19 on stroke care. International Journal of Stroke. julio de 2021;16(5):573-84.

41. Kerleroux B, Fabacher T, Bricout N, Moïse M, Testud B, Vingadassalom S, et al. Mechanical Thrombectomy for Acute Ischemic Stroke Amid the COVID-19 Outbreak: Decreased Activity, and Increased Care Delays. Stroke. julio de 2020;51(7):2012-7.

42. Schroeder EB, Rosamond WD, Morris DL, Evenson KR, Hinn AR. Determinants of Use of Emergency Medical Services in a Population With Stroke Symptoms: The Second Delay in Accessing Stroke Healthcare (DASH II) Study. Stroke. noviembre de 2000;31(11):2591-6.

43. Medoro I, Cone DC. An Analysis of EMS and ED Detection of Stroke. Prehospital Emergency Care. 4 de julio de 2017;21(4):476-80.

44. McCormick N, Bhole V, Lacaille D, Avina-Zubieta JA. Validity of Diagnostic Codes for Acute Stroke in Administrative Databases: A Systematic Review. Quinn TJ, editor. PLoS ONE. 20 de agosto de 2015;10(8):e0135834.

# Anexo: Escala Madrid Direct

| Item | Puntuación |
| --- | --- |
| Sistema motor: BRAZO | 0: Vence gravedad aunque sea con ayuda 1: No vence gravedad |
| Sistema motor: PIERNA | 0: Vence gravedad aunque sea con ayuda  1: No vence gravedad |
| Mirada conjugada | 0: Normal 1: Desviación conjugada de la mirada a un lado |
| Respuesta a órdenes o Reconocimiento del déficit | 0: Normal  1: No obedece órdenes  O no reconoce su debilidad o su lado débil |
| Tensión arterial sistólica | 0: Menos de 180  -1: 181-190  -2: 191-200  -3: 201-210  -4: 211-220 |
| Edad | Si comorbilidad, -1 por cada año que supere los 85 |
|  | Si TOTAL > 1 traslado DIRECTO a centro con trombectomía |

**NOTAS:**

- En los ítems motores, se puntúa sólo aquella extremidad que no consiga vencer gravedad (un balance muscular de 0 a 2 o una puntuación en la escala NIHSS de 3 o 4 en este ítem)

- La desviación de la mirada se puntúa ya sea parcial o forzada (NIHSS de 1 o 2 en este ítem)

- La respuesta a órdenes se puntúa si no obedece la mitad o más de órdenes sencillas (NIHSS de 1 o 2 en este ítem)

- El reconocimiento del déficit se evalúa preguntando al paciente “¿de quién es este brazo?”, o “¿es suyo este brazo?” y adicionalmente “¿puede mover bien los brazos?”. Si cualquiera de las dos respuestas es incorrecta o el paciente muestra otros signos muy evidentes de no reconocer su déficit, se puntuará 1. En este caso, no se evalúa la extinción visual o sensitiva

- La respuesta a órdenes y el reconocimiento del déficit son ítems mutuamente excluyentes, pues es necesario obedecer órdenes para responder a las preguntas de reconocimiento

- En caso de pacientes cuya puntuación sea <2 debido exclusivamente a su edad, y su situación basal sea excelente, se podrá valorar con el neurólogo del hospital de guardia para TM la posibilidad de traslado directo.

- La escala Madrid DIRECT positiva (2, 3 ó 4) indica traslado directo a un centro con capacidad para realizar trombectomía mecánica.
