# Supplementary material for: The COVID-19 pandemic effect on the prehospital Madrid stroke code metrics and diagnostic accuracy
Source: PLoS One. 2022 Oct 10;17(10):e0275831. doi: 10.1371/journal.pone.0275831 (PMC9550046; doi:10.1371/journal.pone.0275831)
Supplement: Attachment — Submitted filename: Response to reviewers v3.docx [file pone.0275831.s005.docx]

Dear editors,

Many thanks for giving us an opportunity to revise our findings. We are particularly grateful to the reviewers for their time, effort and constructive feedback. We have taken their suggestions on board and revised the paper accordingly. Please see our point-by-point responses to their comments below.

Manuscript ID: PONE-D-22-10142

**Tittle of paper:** “The COVID-19 pandemic effect on the prehospital Madrid Stroke code metrics and diagnostic accuracy”.

| Answer | Response |
| --- | --- |
| **Journal Requirements:** | |
| When submitting your revision, we need you to address these additional requirements.  1. Please ensure that your manuscript meets PLOS ONE's style requirements, including those for file naming. The PLOS ONE style templates can be found at  <https://journals.plos.org/plosone/s/file?id=wjVg/PLOSOne_formatting_sample_main_body.pdf> and  <https://journals.plos.org/plosone/s/file?id=ba62/PLOSOne_formatting_sample_title_authors_affiliations.pdf> | The style was updated according to PLOS ONE´s requirements |
| 2. Thank you for stating the following in the Competing Interests section:  I have read the journal's policy and the authors of this manuscript have the following competing interests: Nicolás Riera-López and Jorge Rodríguez-Pardo de Donlebún have received payments for training courses from the Angels Initiative (Boheringer Ingelheim). The other authors do not report any conflicts of interest.  Please confirm that this does not alter your adherence to all PLOS ONE policies on sharing data and materials, by including the following statement: "This does not alter our adherence to  PLOS ONE policies on sharing data and materials.” (as detailed online in our guide for authors <http://journals.plos.org/plosone/s/competing-interests>).  If there are restrictions on sharing of data and/or materials, please state these. Please note that we cannot proceed with consideration of your article until this information has been declared.  Please include your updated Competing Interests statement in your cover letter; we will change the online submission form on your behalf. | Our competing interest have been updated adding the sentence:  *“This does not alter our adherence to PLOS ONE policies on sharing data and materials”.*  As requested for the editor, we want to clarify that data belongs to our Regional Health Authority, and each of the participant hospitals. Spanish law requires permission to be obtained from each of the data protection delegates to share data with third parties.  Restrictions and updated competing interests update, are added in the cover letter. |
| 3. Please note that in order to use the direct billing option the corresponding author must be affiliated with the chosen institute. Please either amend your manuscript to change the affiliation or corresponding author, or email us at [plosone@plos.org](mailto:plosone@plos.org) with a request to remove this option. | Affiliation has been corrected. |
| 4. We note that you have indicated that data from this study are available upon request. PLOS only allows data to be available upon request if there are legal or ethical restrictions on sharing data publicly. For more information on unacceptable data access restrictions, please see <http://journals.plos.org/plosone/s/data-availability#loc-unacceptable-data-access-restrictions>.  In your revised cover letter, please address the following prompts:  a) If there are ethical or legal restrictions on sharing a de-identified data set, please explain them in detail (e.g., data contain potentially sensitive information, data are owned by a third-party organization, etc.) and who has imposed them (e.g., an ethics committee). Please also provide contact information for a data access committee, ethics committee, or other institutional body to which data requests may be sent.  b) If there are no restrictions, please upload the minimal anonymized data set necessary to replicate your study findings as either Supporting Information files or to a stable, public repository and provide us with the relevant URLs, DOIs, or accession numbers. For a list of acceptable repositories, please see <http://journals.plos.org/plosone/s/data-availability#loc-recommended-repositories>.  We will update your Data Availability statement on your behalf to reflect the information you provide. | Our data is available upon reasonable request because the permission to share the data has not been received from the Health Authority yet.  We clarify this requirement in the cover letter adding the following sentence:  *“Data belongs to the regional Health Authority, and each of the hospitals. Spanish law requires permission to be obtained from each of the data protection delegates. the legal owners of the data (our health authorities) do not allow data to be shared with third parties unless there is express permission from the data controllers*”  Data controller contact information: Ricardo Iglesias (protecciondedatos.sanidad@madrid.org) |
| 5. One of the noted authors is a group or consortium Madrid Stroke Network. In addition to naming the author group, please list the individual authors and affiliations within this group in the acknowledgments section of your manuscript. Please also indicate clearly a lead author for this group along with a contact email address.’ | We updated the individual authors and affiliation according to your instructions, as well as in the supplementary material (S1 File). |
| **Additional Editor Comments:** | |
| Thanks for conducting this study, please consider all given important comments by reviewers in revised version, particularity possible bias | We thank the editor for this appreciation. We reviewed the entire manuscript in accordance with the reviewers' comments, with special emphasis on possible biases. |
| **Reviewer #1:** | |
| **Title:** | |
| OK | Thank you for this comment |
| **Abstract:** | |
| According to the definition of the methodology used in the paper, it seems this is not a quasi-experimental study; because the researcher did not have a role for exposure allocation (COVID-19). | Thank you for your comment. We agree with the reviewer that the researcher did not have a role for exposure allocation and therefore it is an observational study in nature.  We therefore accept this suggestion and make the following changes in the manuscript:  Abstract section (page 3, rows 44-46)adding the sentence*: “****Methods:*** *This was a multicentre interrupted time-series observational study of the cohort of stroke codes of SUMMA 112 and of the ten hospitals with a stroke unit in the Community of Madrid*”  Material and methods section (page 07, rows 108-110) adding the sentence*: “An interrupted time series multicentre observational study was conducted that included the SC cohort of SUMMA 112 and the discharge summary data of the ten hospitals with a Stroke Unit of the Madrid Health Region”* |
| It is important to show the duration of each phase in the context of this section (material and method part of the abstract) | We thank the reviewer for this query that clarify our study.  First group of our study corresponds to the first wave of COVID-19 that lasted 79 days and the second group was 78 days corresponding to the same period of 2019.  We add the dates of start and end of each group in the abstract section (page 3, rows 47-50)in order to include these details;  *“We established two groups according to the date on which they were attended: the first during the dates with the highest daily cumulative incidence of the first wave of the COVID-19 (from February 27 to June 15, 2020), and the second, the same period of the previous year (from February 27 to June 15, 2019)”*  Also, we include this information in the inclusion and exclusion criteria section;(page 8-9 rows 150-155)*“Inclusion criteria: patients meeting SC criteria. The recruitment period was split into the following dates: pandemic group (February 27 to June 15, 2020, 79 days) and pre-pandemic group (the same period of the previous year, February 27 to June 15, 2019, 78 days).* |
| **Introduction** | |
| OK | Thank you |
| **Material and method:** | |
| 1. this study is not a quasi-experimental one as explained above. | Thank you for your question. We reproduce here the same information provided to your comment #1 to the abstract section:  We agree with the reviewer that the researcher did not have a role for exposure allocation and therefore it is true that this is an observational study in nature.  We therefore accept this suggestion and make the following changes in the manuscript:  Abstract section (page 3 rows 44-46) adding the sentence*: “****Methods:*** A multicentre interrupted time-series observational study of the cohort of stroke codes of SUMMA 112 and of the ten hospitals with a stroke unit in the Community of Madrid”  Material and methods section (page 7, rows 108-110), adding the sentence*: “*A interrupted time series multicentre observational study was conducted that included the SC cohort of SUMMA 112 and the discharge summary data of the ten hospitals with a Stroke Unit of the Madrid Health Region*”* |
| 2. I think it is necessary to define the study setting in more detail. This especially true for the very critical patients (who might have died before arriving at the hospital). | We are thankful for your suggestion, and we are in full agreement.  We define the study setting in more detail, describing more precisely hospital transfer of the patient, and the collection of data from the discharge report to the Minimum Basic Data Set Database (MBDS) adding the sentence:  In section Setting and population, (page 8, rows 141-143) we wrote: *“Finally, at hospital discharge, a report is filled in with all the diagnoses and treatments, which are recorded by the Coding Service of each hospital in the Minimum Basic Data Set Dataset (MBDS)”*. |
| 3. As a matter of the fact, I am very concern about the matter of immortal time bias which may easily happen in such studies. | We thank the reviewer for sharing this concern. As it can be seen in Table 1, the pre-pandemic and pandemic populations were very similar in those clinical characteristics related to severity of the clinical condition (i.e. Madrid direct scale) so, we understand that this type of bias did not occur or occurred at a negligible level. Our reasoning is that if both periods included patients with the same severity, the likelihood of being accepted by this immortal time is the same in both periods. This makes that our main outcomes, which are related to times employed in the different processes, would not be affected by this eventual mortality before reaching hospital. Also, it is known that, in case of acute stroke, death occurs in a very small proportion of cases. It is estimated that in the Community of Madrid, overall stroke mortality is around 15%, and prehospital mortality is a short fraction of this. Nevertheless, we agree with your concern and, understanding that this type of bias could occur in a low proportion, we add a sentence at Discussion section (page 20, rows 379-381) (so that it is considered when extracting conclusions:  “*In addition, we cannot rule out the possibility of an immortal time bias, but if it were to arise, we believe it would not be significant given the similarity between pandemic and prepandemic populations*.” |
| 4. Please define the method of estimating each time variable in more detail so that the method of the measurements can be reproduced in future studies. | We appreciate your suggestion, and we are now describing more precisely our method of estimating each time variable.  We added this information at the caption of the Figure 1, in section Variables, page 10, which clarify the method of estimating time variables for dispatch, mobilization, intervention, arrive at hospital and discharge.  *“For the main objective, time elapsed in each of the steps of prehospital care were analysed (Fig 1)”*   a. *112 call: The call is picked up by the 112 operator.* b. *Dispatch: A message with the mission data is sent to the ambulance.* c. *Mobilization: The ambulance starts moving towards the mission address.* d. *Intervention: Ambulance stops upon arrival at the mission address.* e. *Departure to hospital: The ambulance with the patient begins to move towards the hospital.* f. *Arrive at hospital: The ambulance with the patient stops upon arrival at the hospital.* g. *Discharge: The patient is discharged from the hospital (to home or another health center).*   *Times a and b are automatically recorded by the system. Times c, d, e and f are manually recorded by pressing a button on the ambulance's tablet PC. Finally, time g is manually recorded by the MDBS Coding Service with the date and time of the hospital discharge report”* |
| 5. This is true for the diagnostic accuracy and severity status as well. | Thank you for this comment. We clarify that stroke diagnosis was confirmed at the admission to the hospital according to ICD-10 (International Classification of Diseases, 10th revision) adding the next sentence in the abstract section page 3 rows 50-54 of the manuscript. “*To assess the performance of the stroke code, we compared each of the pre-hospital emergency service time periods, the diagnostic accuracy (proportion of stroke codes with a final diagnosis of acute stroke out of the total), the proportion of patients treated with reperfusion therapies, and the in-hospital mortality*.” And also in the Material and method section page 10-11, rows 200-205: *“In the hospital phase, the diagnostic accuracy was calculated as the proportion of SC with a final diagnosis of AS (as defined in S1 Table) out of the total number of records, without distinguishing between ischemic and haemorrhagic stroke”* Codes for acute stroke diagnosis are shown in S1 Table: CODE \| DISEASE; G45 \| Transient cerebral ischaemic attacks and related syndromes; G46 \| Vascular syndromes of brain in cerebrovascular diseases; I60 \| Subarachnoid haemorrhage; I61 \| Intracerebral haemorrhage; I62 \| Other nontraumatic intracranial haemorrhage; I63 \| Cerebral Infarction; I65 \| Occlusion and stenosis of precerebral arteries, not resulting in cerebral infarction; I66 \| Occlusion and stenosis of cerebral arteries, not resulting in cerebral infarction "Degree of severity" is a variable that is not specific to stroke, but to health system managers (such as Medicare). It uses the classification of diagnosis-related groups (DRGs) to estimate the severity of patients based on their comorbidities and complications. It adds information about the population studied in each of the groups. We have not described it in more detail as it is not a main objective of the study. |
| 6. In case of severity index, I think estimation of reliability is necessary in your own study. | We thank the reviewer for this comment. As you can see in the comment above, we explained that the severity index is only a variable that more accurately describes the type of patients we are treating in addition to other more specific variables such as the Madrid Direct stroke scale, so we believe that, for our study, calculating the reliability of this index does not add clinical value. |
| 7. Is it possible to assess the number of repeated cases in each period of the study? This is because that in case of large number of these cases, autocorrelation consideration may be necessary for the analysis | We appreciate this comment in order to clarify this issue.  We have reviewed our data and no repeated data have been found in our registry; therefore, there is no need of considering autocorrelation in our analysis. We include the following sentence in the Results section (page 13, row 234), to further explain this fact on our study population.  *“**No repeated cases were found.*” |
| **Results:** | |
| 8. There are a number of variables which are not addressed in the previous section (e.g., Electrocardiogram, temperature and …). | Thank you for this comment. We added the missing variables to the variables section (page 10, rows 196-209):  *“In the prehospital phase, the following data were collected: age, sex, vital signs (blood pressure, heart rate, respiratory rate, oxygen saturation, glycaemia, temperature, and electrocardiographic rhythm), suspicion of large vessel occlusion (measured with the M-Direct scale), if it was cannulated on an intravenous line, place of patient admission and Glasgow Coma Scale.*  *In the hospital phase, the diagnostic accuracy was calculated as the proportion of SC with a final diagnosis of AS (as defined in S1 Table) out of the total number of records, without distinguishing between ischemic and haemorrhagic stroke. The overall severity status of the patients was also analysed using three parameters: the Charlson comorbidity index with updated weights (which evaluates life expectancy at 10 years, classifying the patient's comorbidity as absent, low or high); the degree of severity and risk of mortality (which predict in-hospital mortality with four degrees of risk, namely, minor, moderate, major or extreme), the reperfusion treatment applied, and in-hospital mortality”* |
| 9. According to the results shown in table 1 (lower mean age in pandemic era), I think it may be necessary to adjust the addressed results according to the age of the patients. | We thank the reviewer for this comment. It´s true that in Table 1 we can find statistically significant differences between the two groups in age. However, we consider that a difference of two years in age, is not clinically relevant, nor has it influenced the findings of the study. However, to verify the impact of such differences in our findings, we analysed the differences in all time variables adjusted for the age of the patients. The results were as follows: Min \| **No adjusted (IC)** \| **Adjusted (IC)**; **112 Call-Transmission** \| 0.4 ( -0.11; 0.91) \| 0.4 (-0.11; 0.88); **Transmission-Mobilization** \| 0.5 (0.33;0.98) \| 0.6 (0.29; 0.95); **Mobilization-Intervention** \| -0.1 (-0.92;0.9) \| -0.01 (-0.81; 0.79); **Intervention-Departure to hospital** \| 4.1 (2.49; 5.93) \| 4.2 (2.48; 5.96); **Departure-arrival the hospital** \| 0.4 (-1.59;2.64) \| 0.1( -2.14; 2.38); **Call-Arrival at the hospital** \| 3.1 (0.16;7.17) \| 3.2(-0.51;7.00); **Length of hospital stay (days)** \| 0.2 (-1.28; 0.91) \| 0.1 (-1.14;1.25) As can be seen, both the estimators and their confidence intervals of the unadjusted and the adjusted time variables differences are closely the same. Therefore, we can conclude that the effect of the pandemic is not confounded by age. Accordingly to this comment, we have added in the manuscript the following sentence in Results section (page 15, rows 2598259): *“These analyses were adjusted by age and did not make any significant difference (data not shown)”* |
| **Reviewer #2** | |
| 1. Were the two groups similar in terms of age, sex, type and severity of stroke? Is the pattern of stroke occurrence in two groups the same? | Thank you for your questions.  Table 1 shows that patients in the pandemic group were slightly younger than those in the prepandemic period although this difference did not confounded our analysis as we explained in our response to comment #8 of reviewer #1. Male and female frequencies in both periods are very similar.  Regarding the severity of the stroke, the Madrid-Direct scale showed no significant differences in the proportion of positives cases between both periods. The other severity scales used (which consider other aspects such as complications, comorbidity, etc.) also showed no statistically significant differences. We found no difference in the pattern of stroke occurrence.  We clarify these aspects in the manuscript in Results section (page 13, rows 242-244), adding the following sentence: “No statistically significant differences were detected in the vital signs collected, in the Charlson comorbidity index, the degree of severity, the predicted risk for mortality or the proportion of positive on the Madrid Direct scale”. |
| 2. The manuscript needs to basic scientific editing. | Thank you very much for your comment that help us to improve our manuscript. We´ve checked the entire manuscript and corrected some formatting, wording and spelling. Please see the tracked changes throughout the manuscript. |
| 3. Is it enough to study only the first wave of COVID-19 to reach such results? Isn't it better to evaluate the actions in the next waves? | Thank you very much for raising this question  Our main objective was to analyse what happened at the worst moments of the beginning of the pandemic, when the impact on health services in general has been the greatest, as compared to a control prepandemic period. Therefore, we have analysed the first wave, when there was no great knowledge of the disease, and organizational measures had been implemented urgently, without having been evaluated.   We tried to clarify this in the Introduction section (page 6, rows 99-104): “The main aim of the study was to compare the periods of time spent in each of the phases of the prehospital SC in the CM before the onset of the pandemic and during the period with the highest incidence of SARS-CoV-2 infection in the first wave.”  Also, in Material and methods section (page 8, rows 146-149) we wrote: “Because the objective is to analyse what happened at the times of highest incidence, the days on which the reported ones were above the median of the daily cumulative incidence during the first wave were selected for the pandemic group”. |
| 4. I think the type of study should be changed to observation before- after study. | Thank you for your question. We reproduce here the same information provided to Reviewer #1’s comment to the abstract section:  We agree with the reviewer that the researcher did not have a role for exposure allocation and therefore it is true that this is an observational study. However, we prefer not to use the reference to before-after study as this type of design is frequently interpreted as a paired cohort which is observed twice, and this is not our case. We think our design is better described as an observational interrupted time series study.  We have made the following changes in the manuscript:Abstract section (page 3 rows 44-46)we added the sentence*: “****Methods:*** *This was a multicentre interrupted time-series observational study of the cohort of stroke codes of SUMMA 112 and of the ten hospitals with a stroke unit in the Community of Madrid*”  Material and methods section (page 7, rows 108-110), we added the sentence*: “An interrupted time series multicentre observational study was conducted that included the SC cohort of SUMMA 112 and the discharge summary data of the ten hospitals with a Stroke Unit of the Madrid Health Region”* |
| 5. The code of ethics should be mentioned. | Thank you for your comment.  We mentioned this code of ethics in Material and methods section (page 7, rows 110-112): “*The protocol was approved by the Drug Ethics Committee of the CM (Act 12/2020), which authorized the exemption of informed consent.”* |
| 6. Who answers the calls? Was the experience and skill of the respondents the same? It must be mentioned in the methodology. | Thank you for your questions.  SUMMA 112 call reception is done in two steps. The first person, the telephone operator, collects basic data from the place of the incident and gathers the main symptom in a logical tree. The call is then passed to a physician who conducts a structured interview and provides initial instructions to the patient. If the symptoms are recognized, an advanced ambulance (with emergency physician) is dispatched […]  All first responders have received specific training in acute stroke care as well as in other types of time-dependent pathologies.  For a better understanding of the study we include an extended description of the call management in the Material and methods section; Setting and population (page 7-8 rows 123-143). |
| 7. Hadn't the instructions changed in these two periods? | Thank you for your question.  Instructions for call handling, dispatch, on-site patient management, hospital pre-notification and urgent transfer have not changed during the COVID-19 pandemic. We also clarify this point at the material and method section page 8, row 133:  “*The pre-hospital SC protocol has not changed during the two periods of the study.”* |
| 8. The inclusion criteria for patients in unclear? | Thank you for your suggestion. We rewrote the inclusion criteria section to make it simpler and more understandable and we also introduce a sentence with the exclusion criteria. Please see changes at page 8-9, rows 150-157.  “*Inclusion criteria: patients meeting SC criteria. The recruitment period was split into the following dates: pandemic group (February 27 to June 15, 2020, 79 days) and pre-pandemic group (the same period of the previous year., February 27 to June 15, 2019, 78 days).*  *Exclusion criteria: patients without Health Identification Number (HIN), or without a record in the MBDS.* “ |
| 9. It is better to write abnormal instead of non-normal. | We thank the reviewer for this suggestion. To avoid misunderstandings, we have rewritten the sentence and instead of referring to “non-normal variables” we refer now to “variables that do not follow a normal distribution”. We have changed the correspo0nding sentence (page 11, rows 213-217):  *“Categorical variables are presented as absolute and relative frequencies, quantitative variables with a normal distribution are presented as means and standard deviations, and variables that do not follow a normal distribution are presented as medians and interquartile ranges”* |
| 10. Why is the bootstrap method used to calculate the differences? and why has it been resampled 800 times? Which bootstrap method is used? Parametric or non-parametric? Be clearly mentioned. | Thank you very much for raising this question.  We used a non-parametric bootstrap technique because it is the appropriate method for calculating the confidence intervals of the difference of the medians of the two groups (pandemic and pre-pandemic). According to Efron´s method, confidence intervals can be estimated satisfactorily when there are more than 50 resamples so we carried out 800 to obtain accurate confidence intervals.  For a better description of the methods used, we rewrote the material and method section (page 11, rows 218-224):  *“To evaluate the magnitude of the pandemic effect we calculated the difference between medians of the pre-pandemic and pandemic groups. The confidence intervals for these differences in medians were estimated using non-parametric bootstrap techniques with 800 resamples [28].”*  We have included a new reference in bibliography:  *[28] “B. Efron and R. Tibshirani. Bootstrap methods for standard errors, confidence intervals, and other measures of statistical accuracy. Statistical Sci, 1:54–77, 1986.”* |
| 11. The P<0.01 is not valid. It should be written up to three decimal places or as p<0.001. | We thank the reviewer for this suggestion. We have reported all p-values with three decimal places. |
